# Age Differences in Occupant Motion during Simulated In-Vehicle Swerving Maneuvers

**DOI:** 10.3390/ijerph17061834

**Published:** 2020-03-12

**Authors:** Valentina Graci, Ethan Douglas, Thomas Seacrist, Jason Kerrigan, Julie Mansfield, John Bolte, Rini Sherony, Jason Hallman, Kristy Arbogast

**Affiliations:** 1 Center for Injury Research & Prevention, Children’s Hospital of Philadelphia, Philadelphia, PA 19146, USA; douglase1@email.chop.edu (E.D.); seacrist@email.chop.edu (T.S.); arbogast@email.chop.edu (K.A.); 2 Center for Applied Biomechanics, University of Virginia, Charlottesville, VA 22911, USA; jrk3z@virginia.edu; 3 Injury Biomechanics Research Lab, Ohio State University, Columbus, OH 43210, USA; Julie.Mansfield@osumc.edu (J.M.); John.Bolte@osumc.edu (J.B.); 4 Collaborative Safety Research Center, Toyota Motor Eng. & Mfg. NA, Inc., Ann Arbor, MI 48108, USA; rini.sherony@toyota.com (R.S.); jason.hallman@toyota.com (J.H.); 5 Perelman School of Medicine, University of Pennsylvania, Philadelphia, PA 19104, USA

**Keywords:** pre-crash maneuver, child occupant, muscle activity, occupant kinematics, head displacement, trunk displacement, lateral acceleration, restraints

## Abstract

Background: With active safety and automated vehicle features becoming more available, unanticipated pre-crash vehicle maneuvers, such as evasive swerving, may become more common, and they may influence the resulting effectiveness of occupant restraints, and consequently may affect injury risks associated with crashes. Therefore, the objective of this study was to quantify the influence of age on key occupant kinematic, kinetic, and muscular responses during evasive swerving in on-road testing. Methods: Seat belt-restrained children (10–12 years old), teens (13–17 years old), and adults (21–33 years old) experienced two evasive swerving maneuvers in a recent model sedan on a test track. Kinematics, muscle activity, and seat belt load distribution were determined and analyzed. Results: Compared to teens and adults, children showed greater head and trunk motion (*p* < 0.03), but similar muscle activation in the into-the-belt direction of swerving. In the out–of-the-belt direction, children showed head and trunk motion more similar to teens and adults (*p* < 0.02), but with greater muscle activation. Conclusions: Children showed different neuromuscular control of head and trunk motion compared to older occupants. This study highlights differences in the relationship between kinematics and muscle activation across age groups, and provides new validation data for active human body models across the age range.

## 1. Introduction

Previous research has shown that approximately 80% of drivers perform a critical evasive maneuver before a crash [1]. These pre-crash maneuvers are often low-acceleration, time-extended (LATE) events that result in changes in the occupant state (including position, posture, muscle tensing) that can displace occupants away from idealized seating positions within the restraints [2]. Additionally, as active vehicle technologies come to fruition, we can expect that automated vehicle maneuvers that occur prior to a crash may result in a more frequent displacement (or greater magnitude of displacement) of the occupant state that will further challenge the occupant protection offered by vehicle restraints [2]. Understanding kinematics and muscle response in these loading conditions is particularly important. Kinematics provides quantitative information that is useful in the design of advanced restraint systems. Muscle activation has an important influence on occupant kinematics in the pre-crash phase due to longer duration and lower loads than in crash events.

Evasive swerving is a common pre-crash maneuver characterized by lateral acceleration that may affect the resulting torso location prior to crash loading [3,4]. Torso location has been connected to head injury causation for rear-seated restrained children due to torso rollout from the shoulder belt and head contact with the seat back, door, or B-pillar [3,5,6]. In order to quantify human kinematics and muscle response in evasive swerving maneuvers, there have been several on-road and laboratory studies with human volunteers and anthropomorphic test devices (ATDs). However, many of the previous on-road evasive maneuver studies focused on adult drivers rather than passengers, e.g., [7,8,9]. The situational awareness and the geometrical boundary conditions of the driver seat position vary significantly as compared to rear-seated passengers so that the responses cannot be generalized across the seat positions. Several studies characterized the biomechanical behavior of adult passengers in an actual vehicle environment, e.g., [10,11,12,13]; however, biomechanical and neurophysiological differences between adults and children limit the translation of these findings to younger passengers. Fewer studies have examined the motion of child and teen occupants. Bohman et al. [3] were the first to utilize pediatric human volunteers aged 4–12 years as restrained rear-seated occupants: a single sharp turn was studied with no change in direction as might be expected in emergency swerving. The results of this study quantified pre-impact postures of children and seat-belt slippage as a result of vehicle swerving maneuvers for a variety of restraint systems. Similar testing methods were used by Baker et al. [14] to quantify occupant kinematics and understand restraint interaction among pediatric subjects utilizing two different booster seat designs. Results of the abovementioned studies examined the influence of seat belt position on the potential for torso rollout from the shoulder belt induced by a maneuver characterized by lateral acceleration; however, the reverse acceleration pulse (movement into the shoulder belt) as might be experienced during an extended evasive swerving event was not explored. Furthermore, muscle activity was not explored in these studies.

More recent investigations have examined both kinematics and muscle activation in child and teen occupants compared to adults [15,16,17,18]. Holt et al. [16] quantified occupant kinematics and electromyography (EMG) activity in passengers exposed to sled-simulated evasive swerving maneuvers. These studies highlighted the potential benefit of advanced restraint technology (i.e., a pre-pretensioner) in reducing out-of-position occupant kinematics in pre-crash events characterized by lateral acceleration. Holt et al. also showed that, although there were no statistically significant age differences in peak lateral head and trunk excursion during evasive swerving, there were differences in muscle activation strategies between age groups. Young occupants, particularly teenagers, showed greater muscle activation with greater variability between subjects in the sternocleidomastoid and middle trapezii compared to adults. Graci et al. [15] found that child and teen occupants showed greater muscle activity in the neck muscles during emergency braking maneuvers compared to adults despite lack of statistical significant differences in kinematics between age groups. Booster-seated children showed greater muscle activity in the arms and in the abdominal muscles rather than in the neck muscles compared to non-booster seated children in evasive swerving maneuvers [15,17]. Overall, previous findings suggest that young occupants may use a different muscle activation strategy to achieve similar head and trunk excursion as adult occupants. However, it has not yet been reported how non-booster-seated children, teens and adults react to the same evasive swerving in-vehicle maneuvers booster-seated children were exposed to by Graci et al. [17].

In summary, while there has been meaningful examination of human volunteer kinematics and muscle activity in response to pre-crash maneuvers, substantial gaps in knowledge remain. There are limited data for the passenger seat position, particularly rear rows, data for children or adolescents, and quantitative information about the neuromuscular control of bracing. Therefore, in this study, we quantified the kinematic and muscle activity responses of children, teens, and adults in lateral loading with a change in direction (i.e., a slalom maneuver) in a vehicle environment to improve the relevance of the data to the real world.

## 2. Materials and Methods

### 2.1. Particpants

Seventeen healthy participants (Table 1) without neuromuscular or musculoskeletal conditions or previous injury were enrolled. Height and weight inclusion criteria were based on ranges related to the Center for Disease Control and Prevention (CDC) growth charts [19] and ATD size to capture the range of adult and child sizes typical of rear seat occupants. Only male participants were selected to avoid introducing gender differences as a potentially confounding factor [20,21].

### 2.2. Experimental Procedure

The experimental testing consisted of two phases. In the first phase, the vehicle dynamics were tested with a professional driver only to establish the appropriateness of the test sequences for human volunteers and the repeatability of the maneuver examined in this study. In the second phase, the human subjects testing was performed. The vehicle maneuver tests were conducted with a recent model year four-door sedan at the Vehicle Dynamic Area (VDA) of the Transportation Research Center (TRC) Inc. (Marysville, OH, USA).

**Figure 1:**
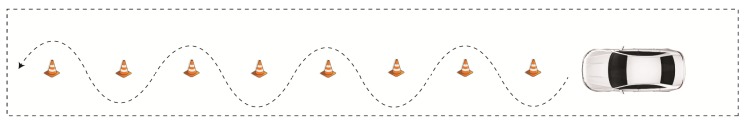
Scheme of the slalom maneuver performed by the vehicle.

The maneuver characteristics were based on the previous literature [3,10] and on the preliminary tests performed with a professional driver on the VDA at TRC. In order to simulate evasive swerving, a slalom maneuver was performed with an average peak lateral acceleration of ~ 0.75 g achieved by having the vehicle move at 65 km/h set via cruise control around a set of 8 cones placed at the distance of 20 meters apart. The slalom consisted of 4 cycles (Figure 1).

All participants were seated in the right rear seat of the vehicle. Before performing the maneuvers, a muscle activity baseline was established by a static trial. In the static trial, participants were instructed to sit in the vehicle in a normal non-tensed posture, with feet on the floor and hands in their lap looking straight ahead for five seconds. After the static trial, each participant remained in the vehicle for a baseline drive where the vehicle was driven on a straight path for approximately 120 m at approximately 50 km/hr. This baseline drive was performed to familiarize the participants, in particular the children, with the vehicle setting. After the baseline drive, each maneuver described above was performed twice for each participant. Each participant was not aware of the exact time at which the maneuver was to occur. Each participant was instructed to sit with feet on the floor and hands in his lap in a non-tensed posture for initial position and act spontaneously during the maneuver as one would do in a real crash-avoidance situation. A brief break of approximately five minutes followed each repetition. The same professional driver conducted the maneuvers for each participant.

### 2.3. Instrumentation

**Figure 2:**
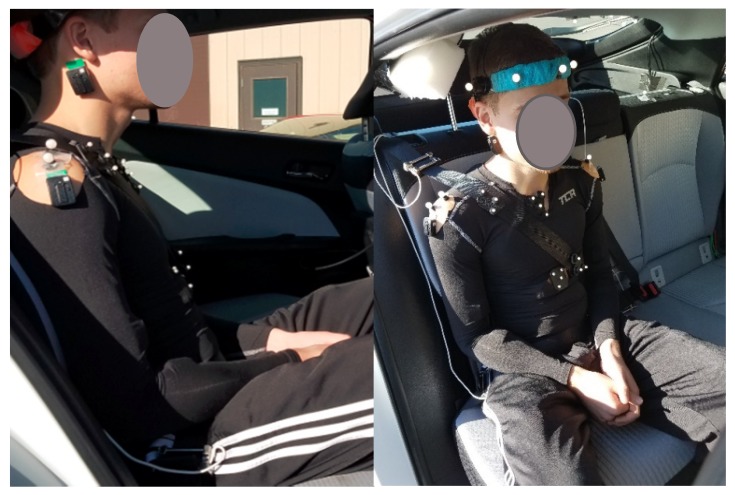
Subject instrumentation: photo-reflective markers placed on the head, the trunk, and the seat belt (left), and wireless EMG (electromyography) sensors visible on the neck and the right deltoid (right).

Vehicle dynamics were measured with an Inertial and Global Positioning System (GPS) measurement unit (Oxford RT 3003, Oxford Technical Solutions Ltd., Oxford, UK) connected to a data acquisition system (Somat eDAQlite, HBM, Inc., Marlborough, MA, USA) placed in the vehicle trunk. The data acquisition system sampled data from the navigation system and the three seat belt load cells (shoulder belt, each side of the lap belt) (Measurement Specialties, TE Connectivity, Inc., Aliso Viejo, CA, USA) at 200 Hz. The right rear seat position was instrumented with an 8-camera infrared 3D motion capture system (Optitrack, NaturalPoint, Inc., Corvallis, OR, USA) with sampling frequency of 200 Hz. The right front seat was moved to the full forward position to leave sufficient space for a compression pole on which the cameras were mounted. Photo-reflective markers were placed on each participant’s head (on a tightly fitted head piece with the markers on the forehead, two on the temple and one on the head top) and trunk (bilateral acromion, suprasternal notch, and xiphoid process), and on the shoulder seat belt (one close to the shoulder area and one on the trunk area). The markers on the seat belt, suprasternal notch, and xiphoid process consisted of an array of four markers placed on rigid structures (Figure 2). Electromyography (EMG) (Trigno EMG System, Delsys Inc., Natick, MA, USA) sensors were placed bilaterally on deltoids, brachioradialis, biceps, rectus femori, rectus abdomini, middle trapezii, and sternocleidomastoids (SCM). These muscles were selected as they were hypothesized to be the most involved in the bracing behavior. Muscle activity data were collected at 2000 Hz.

### 2.4. Data Processing and Analysis

All data processing and analyses were performed with custom Matlab (MathWorks 2015, Inc., Natick, MA, USA) programs. Vehicle acceleration was filtered with a zero-lag 2nd order low pass Butterworth filter with the cut-off frequency set to 6 Hz. Vehicle acceleration bias was removed by subtracting the mean for 0.5 s before the maneuver initiated. Vehicle acceleration profiles from each trial were averaged, and the standard deviation was calculated to examine repeatability of the maneuver. Motion capture data were processed in Motive 2.0 (Optitrack, NaturalPoint, Inc., Corvalis, OR, USA). Head and trunk positions were defined as the geometric centers of the groups of markers placed on the head and the suprasternal notch rigid bodies, respectively. For the head, the rigid-body center approximated the geometric center of the head. Head and trunk positions were filtered with a moving average method spanning five frames. The initial position of the head and the trunk were defined as their average positions for one second prior to the maneuver. The initial position was subtracted from the head and trunk displacements measured during the maneuver. Head and trunk positions were first analyzed non-normalized, and were then normalized by seated height. The primary kinematic outcome measures were the lateral peak head and trunk maximum displacements for each slalom cycle, both into-the-belt (outboard) and out-of-the-belt (inboard).

The raw EMG signals were filtered with a band-pass filter (20–500 Hz, filter order: 558) based on the finite impulse response (Kaiser window method) filter [22]. A root-mean-squared (RMS) method with a 200 ms moving average smoothing window was applied. EMG signals during the maneuver were normalized by the average EMG signal during the static trial. Therefore, muscle activity during the maneuver was expressed as a percentage of the rest, with the rest defined as the muscle activity during the static trial. After the mean EMG was calculated over the duration of each turn for each muscle and for each trial, the data were checked for the presence of outliers, and all data points greater than three standard deviations above the mean were removed. Seat belt forces were filtered by an eight-pole Butterworth filter (Somat TCE, HBM, Inc., Marlborough, MA, USA), and the mean force for 0.5 s before the maneuvers was subtracted from the force signal. The average seat belt loads (shoulder belt, left and right lap belts) were calculated for each swerve of each trial.

Two-way repeated measures and mixed ANOVAs were performed to examine the influence of age (children vs. teens vs. adults) and repetition (first vs. second) on the kinematic outcome measures of displacement (non-normalized and then normalized by seated height). The Tukey’s post-hoc test was used for multiple comparisons. *P*-level was set at 0.05.

## 3. Results

Lateral peak head and trunk displacement out of the belt did not show any effect of age either in analyzing the raw data or when normalized to seated height (*p* > 0.19). Lateral peak head displacement into the belt was greater in children compared to adults (*p* < 0.03), and when normalized, lateral peak head and trunk displacement into the belt were greater in children compared to adults and teens (*p* < 0.05) (Table 2, Figure 2).

Both raw and normalized lateral peak head displacement out of the belt were greater in cycle 4 compared to 1 and 2 (*p* < 0.02). Both raw and normalized lateral peak trunk displacement into the belt were greater in cycles 2, 3 and 4 compared to cycle 1 (*p* < 0.007) (Table 3).

Both raw and normalized lateral peak head displacement out-of-the-belt were greater in repetition 1 compared to 2 (*p* < 0.006). Raw lateral peak head displacement into-the-belt and trunk displacement out-of-the-belt were greater in repetition 1 compared to 2 (*p* < 0.02) but not when normalized to seated height (*p* > 0.09), (Table 4).None of the interaction effects between factors were statistically significant (*p* > 0.06).

**Figure 3:**
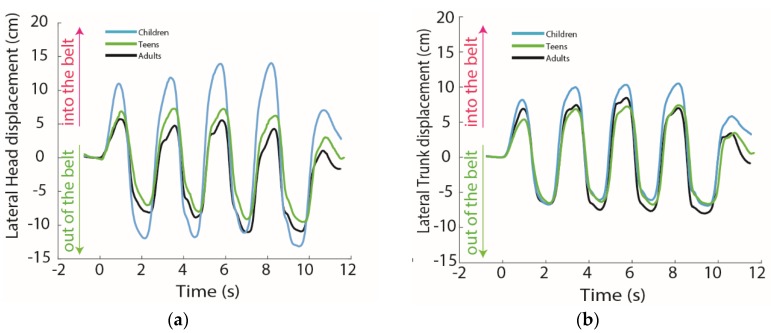
Time series of head and trunk displacement averaged for each age group. Graphs with standard deviations are reported in the Appendix A but not on this graph for visual clarity. (**a**) Lateral head displacement (cm); (**b**) Lateral trunk displacement (cm).

**Figure 4:**
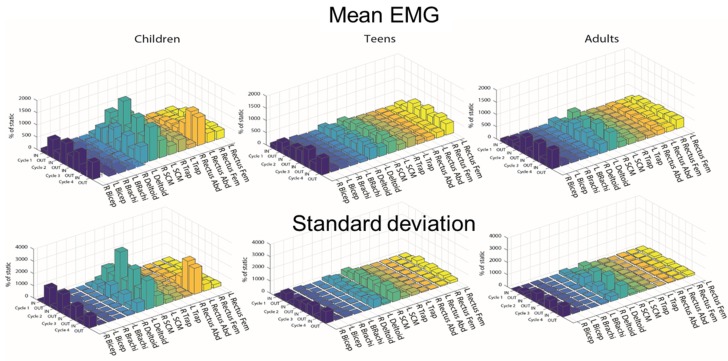
Mean and SD of normalized to static trial Mean EMG calculated for each cycle and age group and expressed as a percentage of muscle activation in the static trial. SCM: sternocleidomastoids; EMG: electromyography.

Mean EMG generally showed greater muscle activation in children compared to teens and adults, with greater variability as well (Figure 3). In particular, the right and left SCMs, left deltoid, and right bicep showed greater activation with greater variability in all age groups compared to other muscles (Figure 4), although children showed greater activation in these muscles compared to other age groups.

The seat belt loads in the into-the-belt direction were smaller than 25 N and are not reported. For the out-of-the-belt direction, adults showed greater maximum shoulder seat belt loads compared to children and teens (*p* < 0.03) (Table 5). No age differences were detected in lap belt loads. Overall, the maximum seat belt load out of the belt of the shoulder, right lap and left lap belts tended to increase with the number of cycles (Table 6). Only the maximum load of the right lap belt showed a significant effect of repetition with greater load in repetition 1 compared to repetition 2 (Table 7).

## 4. Discussion

The aim of this study was to characterize age differences in head and trunk kinematics and muscle activation in seat belt-restrained rear-seated children as compared to teenager and adult rear-seated occupants.

In the motion out of the belt, children, teens, and adults showed no difference in head and trunk excursion, including when head and trunk excursion was normalized to seated height. While in into-the-belt motion, children had greater head and trunk displacement than teens and adults. Head and trunk excursion also increased across the swerving maneuver.

This finding in the kinematics, when interpreted together with muscle activation during the maneuver, suggests that children have a different neuromuscular control of head and trunk motion compared to older occupants when bracing during a slalom maneuver. In the motion of the occupant into the belt, children showed similar muscle activation (Figure 4) to teens and adults, but greater lateral peak head and trunk displacement than adults and teens. In the motion out of the belt, however, children showed similar head and trunk motion to older occupants, but greater neck and right arm muscle activation. This suggests that children performed greater muscle activation than adults and teens to achieve similar head and trunk displacement, although the timing of this activation was not investigated. The differences in neuromuscular strategies of bracing between age groups is the main novelty of this study.

All age groups showed activation of similar muscles during the maneuver: the right bicep, the left deltoid, the SCMs, and the rectus femoris. In adults and teens, the neck muscles show more activation compared to other muscles overall. This is in line with other studies that examined muscle activation during pre-crash maneuvers and found that neck muscles are particularly active during emergency braking [12,15]. The activation of the right and left SCMs does not seem modulated by the into-the-belt and-out-of-the-belt motion, but it is rather bilateral overall (Figure 4). Therefore, it is plausible that part of this muscle response is due to the startle reflex superimposed on the postural response triggered by the vehicle acceleration.

Neuromuscular control of head and trunk motion changed with cycles as well: in the later cycles, head and trunk motion increased across all age groups, suggesting that occupants fine-tuned their strategy to control head and trunk motion over the duration of the maneuver. Particularly, in the out-of-the-belt direction, the arms (deltoids and biceps) and the neck muscles (SCMs) showed less activation over time (Figure 4), while the seat belt load increased with cycles (Table 6). By becoming more comfortable with the maneuver, occupants may have relied more on the seat belt in the later cycles rather than on bracing by contracting their arm and neck muscles. However, the seat belt load was generally small (Table 6 and Table 7), in line with the low acceleration nature of the maneuver.

Similarly to the laboratory-based study of Holt et al. [16], we found no age differences in kinematics in the out-of-the-belt direction. However, Holt et al. [16] registered greater peak lateral head and trunk excursion than in our study. Our findings also differ in the into-the-belt motion. According to Holt et al. [16], children showed smaller head displacement (8 cm) in the into-the-belt direction compared to teens (14–16 cm). In our study, children showed greater head motion in the into-the-belt direction (14 cm) compared to teens (8 cm) and adults (6 cm) (Table 2). These differences could have been due to several reasons. It is plausible that the dissimilarities between the in-vehicle environments versus the laboratory setting contributed to the differences in results. Holt et al. [16] used realistic vehicle seats, and the seating compartment did not have a vehicle roof and a vehicle door. Geometrical structures of the vehicle door and roof could have influenced the motion in the into-the-belt direction of the occupants in our study. The “into-the-belt” motion in our study also means “into the door trim and roof line of the vehicle”; since teens and adults were taller than children, their motion may have been more influenced by the vehicle geometry than the children’s motion.

An evasive maneuver simulated in a vehicle, which would also add a forward component to the loading profile, could influence kinematics. It is possible that, compared to the study by Holt et al. [16], the combined loading in the current study was more difficult to decode by a child’s vestibular system and led to greater head and trunk motion. Another possible explanation of the differences between the two studies is that our slalom maneuver would start with an occupant moving into the belt, while in the study by Holt et al. [16], the slalom maneuver would start with an occupant moving out of belt. The first swerve that an occupant experiences is the most unexpected, and the first swerve was in different directions in the two studies. This could have influenced the occupant bracing strategy to the subsequent swerves.

Ghaffari et al. [13] also exposed adult male occupants to an evasive swerving maneuver entailing only two swerves. The authors analyzed only the motion out of the belt, and the lateral peak head and trunk displacement was slightly greater than the one found in our study. According to Ghaffari et al. [13], head excursion was 15 cm, and trunk excursion was 13 cm, while in the current study, it was 12 cm and 8 cm, respectively. It is plausible that differences in study set-up could have led to the slight differences in kinematic magnitudes. For example, in our study, the occupants underwent eight swerves, while in the study by Ghaffari et al. [13], the occupants underwent only two swerves. Regardless of the differences in head and trunk excursion between the two studies, similar results in relation to muscle activation in adult occupants were found [12]. Adult occupants showed greater activation in the neck muscles compared to most of the other muscles during the maneuver, however, in both studies, adults show low muscle activation overall. The novelty of our study is that children showed greater muscle activation overall, particularly in the neck muscles, compared to older occupants (Figure 4).

Muscle activation data can enhance the potential of human body models (HBMs) to be valuable tools to enable the study of this pre-crash phase. A number of studies have focused on adding muscles and neuromuscular control [23,24] to the models. These active HBMs implement posture maintenance and reflexive responses by means of position feedback control and co-contraction of muscles to simulate occupant bracing. The data to develop and validate these models come from volunteer responses to pre-crash events in on-road or laboratory tests, however, the data are limited across a broad range of important parameters, such as age, body habitus, loading conditions, and vehicle/restraint designs. This study provides useful data that can enrich the current HBMs.

Our study presents some limitations. The maneuver was performed in a single vehicle environment, and the generalizability of these results to other vehicle interior geometries has not been investigated. Although we have used an actual vehicle environment, the testing site was not fully naturalistic, as the study was conducted with instrumentation on a test track, and not in a real traffic situation; this was mitigated by participants being generally unaware of the timing of the maneuvers. Evasive swerving maneuvers may realistically be shorter than the one simulated for this study, however, in order to fully capture the real difference in neuromuscular control between age groups without the influence of a startle reflex due to a quick change in acceleration, we needed a longer maneuver. It is plausible that other muscles besides those measured in this study may have contributed to participants’ motion, such as deep muscles that are unable to be detected by surface EMG. Muscle activity was also not normalized to maximum voluntary isometric contraction (MVIC), but to the rest [17], and therefore may be noisier than MVIC-normalized data. Subcutaneous EMG and MVIC assessments were not utilized because of the challenge of testing children and minimizing test time. Another limitation of the study is the relatively small sample of participants per group. Although the age groups were small, we were able to detect statistically significant age differences in the kinematics. Previous studies have been able to detect statistical differences with equally small samples of participants [15].

## 5. Conclusions

Head and trunk kinematics in simulated evasive swerving maneuvers were characterized for three different age groups. Children had greater displacement than their older counterparts in the into-the-belt direction, while in the out-of-the-belt direction, there were no age differences in kinematics. Kinematics increased across the swerving maneuver so that the last of four cycles produced the greatest displacement. The main novel finding in this study is that children demonstrated different neuromuscular control of bracing behavior compared to adults and teens: they needed greater muscle activation to achieve similar lateral head and trunk displacement out of the belt during the swerving maneuver. When muscle activation was similar across the age groups, children demonstrated greater lateral head and trunk displacement. These findings suggest that the children’s sensory and neuromuscular systems interpret pre-crash maneuvers differently. These data provide useful quantitative kinematics and muscle responses that can be incorporated into current HBMs. It would be beneficial for these models to incorporate these age-based differences in muscle response when used to estimate kinematics in crash avoidance maneuvers. In this study, we examined an evasive swerving maneuver without braking. Future research directions may include the examination of evasive swerving with both manual and automated emergency braking to explore child and adult kinematics in other types of common pre-crash scenarios.

## Figures and Tables

**Table 1:** Mean (SD) of participants’ age, and relevant anthropometrics.

Age Groups	Subject Number	Age (Years)	Seated Height (cm)	Weight (kg)
Children	7	11.6 (6.2)	76.7 (6.2)	47.8 (12.8)
Teens	8	15.1 (1.2)	84.8 (5.3)	60.3 (8.2)
Adults	9	23.8 (4.8)	88.1 (4.1)	70.5 (10.5)

**Table 2:** Mean (SD) of normalized and non-normalized kinematic outcome measures: effect of age.

Dependent Measures	Children (C)	Teens (T)	Adults (A)	ANOVA *p*-Values	Post-hoc *p*-Values
Lateral peak **head** displacement **out of the belt (cm)**	12.9 (6.05)	9.4 (5.8)	12.3 (4.2)	*p* = 0.25	
Normalized	0.2 (0.08)	0.1 (0.06)	0.1 (0.05)	*p* = 0.19	
Lateral peak **head** displacement **into the belt (cm)**	13.6 (8.3)	7.9 (4.1)	6.1 (3.8)	*p* < 0.03 *	C >A *p* < 0.03 *
Normalized	0.2 (0.1)	0.1 (0.05)	0.1 (0.04)	*p* = 0.007 *	C >T, A *p* < 0.04 *
Lateral peak **trunk** displacement **out of the belt (cm)**	6.7 (3.11)	6.7 (2.8)	7.9 (2.5)	*p* = 0.41	
Normalized	0.09 (0.04)	0.08 (0.04)	0.09 (0.03)	*p* = 0.78	
Lateral peak **trunk** displacement **into the belt (cm)**	10.1 (4.7)	7.1 (2.14)	7.8 (2.5)	*p* = 0.11	
Normalized	0.1 (0.06)	0.08 (0.03)	0.09 (0.03)	*p* < 0.02 *	C >T, A *p* < 0.05 *

* *p* ≤ 0.05.

**Table 3:** Mean (SD) of normalized and non-normalized kinematic outcome measures: effect of cycle.

Dependent Measures	Cycle 1 (c1)	Cycle 2 (c2)	Cycle 3 (c3)	Cycle 4 (c4)	ANOVA *p*-Values	Post-hoc *p*-Values
Lateral peak **head** displacement **out of the belt (cm)**	10.6 (5.8)	10.3 (5.01)	11.9 (5.7)	12.5 (5.6)	*p* = 0.003 *	c4 > c1,2, *p* < 0.009 *
Normalized	0.1 (0.07)	0.01 (0.06)	0.1 (0.07)	0.2 (0.07)	*p* = 0.008 *	c4 > c1,2, *p* < 0.02 *
Lateral peak **head** displacement **into the belt (cm)**	8.4 (4.3)	8.8 (6.06)	9.6 (6.7)	8.5 (7.3)	*p* = 0.37	
Normalized	0.1 (0.06)	0.1 (0.08)	0.1 (0.09)	0.1 (0.09)	*p* = 0.29	
Lateral peak **trunk** displacement **out of the belt (cm)**	6.9 (2.5)	6.9 (2.8)	7.1 (3.06)	7.5 (2.9)	*p* = 0.42	
Normalized	0.08 (0.03)	0.08 (0.04)	0.09 (0.04)	0.09 (0.04)	*p* = 0.11	
Lateral peak **trunk** displacement **into the belt (cm)**	6.9 (2.5)	8.5 (3.5)	8.9 (3.5)	8.3 (3.7)	*p* < 0.001 *	c1 < c2,3,4, *p* < 0.007 *
Normalized	0.08 (0.03)	0.1 (0.05)	0.1 (0.04)	0.1 (0.05)	*p* < 0.001 *	c1 < c2,3,4, *p* < 0.006 *

* *p* ≤ 0.05.

**Table 4:** Mean (SD) of normalized and non-normalized kinematic outcome measures: effect of repetitions.

Dependent Measures	Repetition 1	Repetition 2	ANOVA *p*-Values
Lateral peak **head** displacement **out of the belt (cm)**	12.5 (5.6)	10.2 (5.2)	*p* < 0.001 *
Normalized	0.2 (0.07)	0.1 (0.06)	*p* = 0.006 *
Lateral peak **head** displacement **into the belt (cm)**	9.8 (6.5)	7.8 (5.8)	*p* < 0.02 *
Normalized	0.1 (0.09)	0.1 (0.07)	*p* = 0.28
Lateral peak **trunk** displacement **out of the belt (cm)**	7.7 (2.9)	6.5 (2.6)	*p* = 0.005 *
Normalized	0.09 (0.04)	0.08 (0.04)	*p* = 0.09
Lateral peak **trunk** displacement **into the belt (cm)**	8.8 (3.53)	7.5 (3.08)	*p* = 0.67
Normalized	0.1 (0.05)	0.09 (0.04)	*p* = 0.17

* *p* ≤ 0.05.

**Table 5:** Mean (SD) of the maximum seat belt load: effect of age.

Dependent Measures	Children (C)	Teens (T)	Adults (A)	ANOVA *p*-Values	Post-hoc *p*-Values
Maximum shoulder load out of the belt	40.2 (28.5)	45.9 (30.4)	87.9 (55.3)	*p* = 0.01 *	C,T < A, *p* < 0.03 *
Maximum right lap load out of the belt	48.7 (27.9)	50.8 (14.5)	66.9 (30.3)	*p* = 0.13	
Maximum left lap load out of the belt	24.9 (18.6)	19.39 (7.9)	30.9 (18.1)	*p* = 0.08	

* *p* ≤ 0.05.

**Table 6:** Mean (SD) of the maximum seat belt load: effect of cycle.

Dependent Measures	Cycle 1 (c1)	Cycle 2 (c2)	Cycle 3 (c3)	Cycle 4 (c4)	ANOVA *p*-Values	Post-hoc *p*-Values
Maximum shoulder load out of the belt	45.4 (38.1)	59.4 (52.0)	65.5 (48.1)	73.4 (44.5)	*p* < 0.001 *	c1 < c2,3,4, *p* < 0.02 *, c2 < c4, *p* < 0.04 *
Maximum right lap load out of the belt	67.8 (27.6)	46.8 (16.8)	57.9 (27.1)	58.9 (30.4)	*p* < 0.001 *	c1 < c2,3,4, *p* < 0.02 *
Maximum left lap load out of the belt	13.3 (16.5)	19.3 (29.7)	18.1 (24.6)	20.7 (21.5)	*p* = 0.03 *	c1 < c4, *p* < 0.03 *

* *p* ≤ 0.05.

**Table 7:** Mean (SD) of maximum seat belt load: effect of cycle.

Dependent Measures	Repetition 1	Repetition 2	ANOVA *p*-Values
Maximum shoulder load out of the belt	67.6 (50.7)	65.5 (73.4)	*p* = 0.14
Maximum right lap load out of the belt	59.7 (31.8)	54.3 (21.4)	*p* = 0.04 *
Maximum left lap load out of the belt	27.5 (18.5)	23.2 (13.3)	*p* = 0.08

* *p* ≤ 0.05.

**Figure A1:**
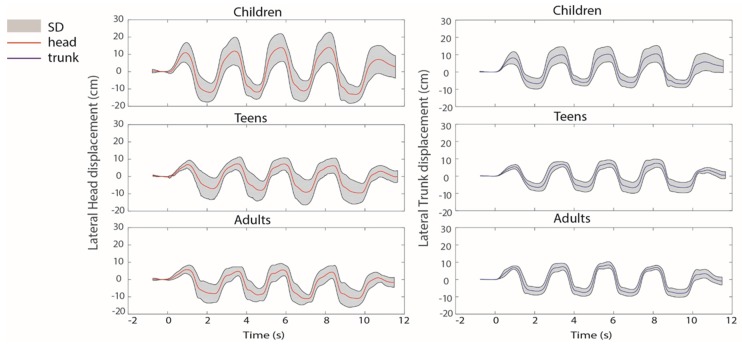
Time series of head and trunk displacement averaged for each age group.
